# Method of Taking Impressions

**Published:** 1903-06-15

**Authors:** 


### METHOD OF TAKING IMPRESSIONS.

The purpose of the method devised by Drs. Latzer and Weisl is essentially to hold together by means of a piece of gauze the different pieces of a plaster impression fractured during its removal from the mouth. A tray of the proper size and shape is selected, and after it has been coated with vaseline its entire surface is covered with a layer of gauze previously soaked in water. The gauze is made to project two or three centimeters beyond the margin and over the sides of the tray. It is not essential that the material should rest smoothly against the surface of the tray, as the presence of folds does not at all interfere with the result. The plaster used in filling the tray should be at first of liquid consistence, so that when it is poured the gauze will be raised slightly. By this plan, when the tray is completely filled the gauze will be contained within the substance of the plaster. The author uses a weave of gauze known as "Mechlin" as it possesses the qualities necessary for this kind of work—namely, thinness combined with strength of fiber. After the tray has been introduced into the mouth it is allowed to remain *in situ* until the plaster has thoroughly hardened. It can then be removed without any difficulty on account of the coating of vaselin which was made before pouring the plaster. The impression is now removed by means of the middle fingers, introduced between the jaw and the cheek. The plaster will be fractured, of course, but it will not come out in too small pieces; and further, not a single piece can be lost. It is claimed for this method that it facilitates the removal of the impression from the mouth without any crumbling of the plaster, and secures the accurate reconstruction of the impression, all the sections being held together by means of the gauze.—*Cosmos.*

